# Epidermal growth factor signaling protects from cholestatic liver injury and fibrosis

**DOI:** 10.1007/s00109-016-1462-8

**Published:** 2016-08-27

**Authors:** Jasmin Svinka, Sandra Pflügler, Markus Mair, Hanns-Ulrich Marschall, Jan G. Hengstler, Patricia Stiedl, Valeria Poli, Emilio Casanova, Gerald Timelthaler, Maria Sibilia, Robert Eferl

**Affiliations:** 1 Institute of Cancer Research, Medical University of Vienna & Comprehensive Cancer Center (CCC), Vienna, Austria; 2 Ludwig Boltzmann Institute for Cancer Research (LBI-CR), Vienna, Austria; 3 Department of Molecular and Clinical Medicine, Institute of Medicine, Sahlgrenska Academy, University of Gothenburg, Gothenburg, Sweden; 4 Leibniz Research Centre for Working Environment and Human Factors at the Technical University of Dortmund (IfADo), Dortmund, Germany; 5 Department of Molecular Biotechnology and Health Sciences, Molecular Biotechnology Center, University of Turin, Turin, Italy; 6 Department of Pharmacology, Center of Physiology and Pharmacology, Comprehensive Cancer Center, Medical University of Vienna, Vienna, Austria

**Keywords:** Epidermal growth factor receptor EGFR, Signal transducer and activator of transcription 3 STAT3, Cholestasis, Bile acids, Liver injury, Hepatocyte apoptosis

## Abstract

**Abstract:**

We have demonstrated that the signal transducer and activator of transcription 3 (STAT3) protects from cholestatic liver injury. Specific ablation of STAT3 in hepatocytes and cholangiocytes (STAT3^∆hc^) aggravated liver damage and fibrosis in the Mdr2^−/−^ (multidrug resistance 2) mouse model for cholestatic disease. Upregulation of bile acid biosynthesis genes and downregulation of epidermal growth factor receptor (EGFR) expression were observed in STAT3^∆hc^ Mdr2^−/−^ mice but the functional consequences of these processes in cholestatic liver injury remained unclear. Here, we show normal canalicular architecture and bile flow but increased amounts of bile acids in the bile of STAT3^∆hc^ Mdr2^−/−^ mice. Moreover, STAT3-deficient hepatocytes displayed increased sensitivity to bile acid-induced apoptosis in vitro. Since EGFR signaling has been reported to protect hepatocytes from bile acid-induced apoptosis, we generated mice with hepatocyte/cholangiocyte-specific ablation of EGFR (EGFR^∆hc^) and crossed them to Mdr2^−/−^ mice. Importantly, deletion of EGFR phenocopied deletion of STAT3 and led to aggravated liver damage, liver fibrosis, and hyperproliferation of K19^+^ cholangiocytes. Our data demonstrate hepatoprotective functions of the STAT3-EGFR signaling axis in cholestatic liver disease.

**Key message:**

- STAT3 is a negative regulator of bile acid biosynthesis.
- STAT3 protects from bile acid-induced apoptosis and regulates EGFR expression.
- EGFR signaling protects from cholestatic liver injury and fibrosis.

## Introduction

Chronic cholestatic liver diseases are characterized by retention of bile acids in the liver which results in alterations of hepatobiliary bile acid transport and enzyme activities participating in bile acid biosynthesis. Hydrophobic bile acids are particularly toxic and promote cholangiocyte/hepatocyte damage, liver fibrosis, cirrhosis, and formation of hepatocellular carcinoma (HCC) under cholestatic conditions [1–3]. Deposition of collagen and other extracellular matrix components is an orchestrated event in cholestatic liver fibrosis and involves several cell types including Kupffer cells and stellate cells. The latter are activated by inflammatory cytokines and responsible for deposition of collagen together with portal myofibroblasts [2]. Despite profound knowledge about cell types that promote cholestatic liver fibrosis and cirrhosis, little is known about hepatoprotective factors that modulate initial events of cholestatic liver injury. The transcription factor STAT3 is required for liver regeneration and hepatoprotection in various chronic liver diseases [4]. STAT3 is mainly activated by IL-6 (interleukin 6) and IL-22 in hepatocytes. These cytokines bind to gp130 (glycoprotein 130) receptors and promote phosphorylation of STAT3 at tyrosine-705 (pY-STAT3) via Janus kinases (JAKs), STAT3 dimerization, and nuclear translocation [4].

Hepatoprotective functions of STAT3 signaling in cholestatic liver disease have been investigated in mice lacking STAT3, IL-6, gp130, or express pathway specific gp130 mutants. Bile duct ligation, cholic acid feeding, and genetic deletion of the Mdr2 gene (Mdr2^−/−^) resulted in aggravated cholestatic liver injury and fibrosis in hepatocyte-specific STAT3 knock-out mice and IL-6^−/−^ mice [5, 6]. Moreover, mice with hepatocyte-specific deletion of gp130 (gp130^hepa^) displayed aggravated liver fibrosis and collagen deposition after DDC (3,5-diethoxycarbonyl-1,4-dihydrocollidine) feeding which is a chemical model for sclerosing cholangitis [7]. The use of specific gp130 knock-in mutant alleles that lack either the region for STAT3 activation (gp130^∆hepaSTAT^) or carry a Y757F mutation that impedes activation of the Ras/MAPK pathway (gp130^∆hepaRas^) [7] demonstrated that the hepatoprotective activity of gp130 signaling was due to gp130-mediated STAT3 activation.

Several cellular and molecular mechanisms might account for the hepatoprotective activity of IL-6/gp130/STAT3 signaling in cholestatic liver injury [4]. We demonstrated that enzymes for bile acid biosynthesis are upregulated in livers of Mdr2^−/−^ mice, lacking STAT3 in hepatocytes and cholangiocytes (STAT3^∆hc^) [6], which might result in increased production of toxic bile and aggravated cholestatic liver damage. Moreover, expression of epidermal growth factor receptor (EGFR) was downregulated in livers of STAT3^∆hc^ Mdr2^−/−^ mice. EGFR signaling protects hepatocytes from bile acid-induced apoptosis which was demonstrated in vitro with hepatocytes harboring a dominant negative ERBB1 allele. A similar protective effect was observed after pretreatment of hepatocytes with the EGFR antagonist Iressa [8–11].

Here, we employed Mdr2^−/−^ mice to functionally test the hepatoprotective function of EGFR signaling in bile acid-induced liver injury and fibrosis. Importantly, conditional inactivation of EGFR in hepatocytes and cholangiocytes (EGFR^∆hc^) of Mdr2^−/−^ mice led to severe jaundice and strongly aggravated liver damage and fibrosis. These data suggest a pivotal hepatoprotective function for EGFR signaling in cholestatic liver disease.

## Methods

### Mice

STAT3^∆hc^ mice were generated by crossing mice carrying floxed alleles of STAT3 [12] to AlfpCre transgenic mice [13]. Furthermore, mice harboring floxed alleles of EGFR [14] were crossed to AlfpCre transgenic mice. Resulting AlfpCre EGFR^flox/flox^ (EGFR^∆hc^) mice were bred with Mdr2^−/−^ mice [15] to generate EGFR^∆hc^ Mdr2^−/−^ mice. Blood sera, bile, and liver tissue of a 7 week old male mouse were used for analyses. All mouse experiments were performed in accordance with Austrian and European laws and with the general regulations specified by the Good Science Practice guidelines of the Medical University of Vienna.

### Histology and immunohistochemistry

Bile canaliculi, sinusoids, and central veins were visualized by immunofluorescence using a combination of antibodies directed against DPPIV/CD26, glutamine synthetase (GS), and secondary fluorochrome coupled antibodies as described previously [16]. For other stainings, livers were formalin-fixed and paraffin-embedded. Hematoxylin/eosin and Sirius-red stainings were performed according to standard procedures. Immunohistochemical stainings were performed with antibodies for CD3 (Histocom, RM-9107), F4/80 (eBioscience, 14–4801-82), Ki67 (Novocastra, NCL-Ki67p), and K19 (monoclonal rat anti-Troma-III antibody provided by Rolf Kemler). For detection of apoptotic cells, ApopTag® Peroxidase in Situ Apoptosis Detection Kit (Millipore, S7100) was used.

### Cell culture, MTT assay, and caspase 3/8 activity assay

Primary hepatocytes were isolated by collagenase liver perfusion using perfusion buffer (17,701–038, Applied Biosystems) and subsequent digestion buffer (17,703–034, Applied Biosystems). Cells were seeded in collagen coated wells and kept in Williams E medium (12,551–032, Applied Biosystems) overnight. On the following day, primary hepatocytes were treated with different concentrations of bile acids. Immortalized hepatocytes were cultured in RPMI supplemented with 80 ng/ml TGF-alpha (T7924, Sigma), 60 ng/ml IGF-II (I2526, Sigma), and 2.8 μM insulin (Novo Nordisk). Before stimulation with IL-6 (40 ng/ml, ImmunoTools, 12,340,065), immortalized hepatocytes were cultured over night without growth factors and treated with IL-6 for 2 h and 4 h. For viability testing, cells were incubated for 3 h at 37 °C with 1 mg/ml MTT (3-(4,5-dimethylthiazol-2-yl)-2,5-diphenyltetrazolium bromide). The plates were dried and the crystals were dissolved. Absorbance was measured at 550 nm to assess cell viability. For detection of active caspases, cells were lysed and incubated with fluorescent substrates specific for caspase 3 (Ac-DEVD-AFC, ALX-260-032, Enzo Life Sciences) or caspase 8 (Ac-LETD-AFC, ALX-260-118, Enzo Life Sciences) for 1 h at 37 °C. Fluorescence was measured at an excitation wavelength of 400 nm and an emission wavelength of 505 nm.

### PCR for genotyping

Genotyping was performed by PCR with primers 5′-gcgtctgactctacaacc-3′, 5′-agcctcatccttaggtact-3′ and 5′-gactgtgataaccttcagtg-3′ for floxed STAT3, 5′-aagtttaagaaaccccgctctact-3′, 5′-gcctgtgtccgggtctcgtcg-3′ and 5′-caaccagtgcacctagcctggc-3′ for floxed EGFR, 5′-cggtcgatgcaacgagtgatgagg-3′ and 5′-ccagagacggaaat-3′ for AlfpCre, 5′-cgagccgaggatgcataagt-3′, 5′-gataagcctgctcgatgcct-3′, 5′-tgtcaagaccgacctgtccg-3′ and 5′-tattcggcaagcaggcatcg-3′ for Mdr2.

### qPCR

Total RNA was isolated with TRIzol (Life Technologies, 15596-018) and reverse transcribed with QuantiTect Reverse Transcription Kit (Qiagen, 205313). qPCR was performed using Fast SYBR Green Mastermix (Thermo Fisher Scientific, 4385616) and an Applied Biosystems 7500 Fast Real Time PCR System with primers 5′-caccctcaagagcctgagtc-3′ and 5′-gttcgggctgatgtaccagt-3′ for COL1, 5′-caggtgaacccggcaagaacg-3′ and 5′-ggggaccagggcgaccact-3′ for COL3, 5′-tcctcttgttgctatcactgatagctt-3′ and 5′-cgctggtataaggtggtctcgtt-3′ for TIMP1, 5′-ccagaagaagagcctgaacca-3′ and 5′-gtccatccagaggcactcatc-3′ for TIMP2, 5′-agaggtcacccgcgtgctaa-3′ and 5′-tcccgaatgtctgacgtattga-3′ for TGFB1, 5′-tcgtccgctttgatgtctca-3′ and 5′-aaatctcgcctcgagctcttc-3′ for TGFB2, 5′-ccgaggactatgaccgggataa-3′ and 5′-cttgttgcccaggaaagtgaag-3′ for MMP2, 5′-atcccaccaaagtgagaacg-3′ and 5′-taatttccctccccggttac-3′ for CTGF, 5′-aatcccaggaccaactatggcagc-3′ and 5′-gaggcaaacttctgttccaatgg-3′ for EGFR, 5′-tgtttgtgatgggtgtg-3′ and 5′-tacttggcaggtttctc-3′ for GAPDH. The expression levels of transcripts were calculated with the comparative CT (threshold concentration) method. The individual RNA levels were normalized for GAPDH and are depicted as relative expression levels.

### Serum measurements

Serum levels of bilirubin, alanine aminotransferase (ALT), and alkaline phosphatase (ALP) were measured using the Reflotron® System (Roche Applied Science).

### Bile flow measurement and bile composition

To measure bile flow, mice were anesthetised and kept on a heating plate during the experiment. The common bile duct was ligated using a string. The gall bladder was punctuated and a cannula was inserted and fixed. The bile was collected in a tube for 30 min, and afterwards the liver weight was measured. Bile acids in the bile were analyzed by isotope-dilution gas chromatography-mass spectrometry (GCMS) as described previously [17].

### Hydroxyproline

Livers were homogenized and hepatic hydroxyproline levels were measured as described previously [18].

### Western blot

Protein lysates were obtained according to standard procedures and analyzed by Western blot with antibodies for P-STAT3 (Cell Signaling, 9145) and β-actin (Sigma, A5316).

### Statistics

Significant differences were calculated with GraphPad Prism 5. Comparisons of the two groups were analyzed with unpaired *t* test or Mann-Whitney test. For more than two groups One-Way Analysis of Variance (ANOVA) and Bonferroni post test or Kruskal Wallis, and Dunns post test were used. Significant differences between experimental groups are stated as: *p < 0.05, **p < 0.01, or ***p < 0.001.

## Results

### STAT3 inhibits bile acid production

**Fig. 1:**
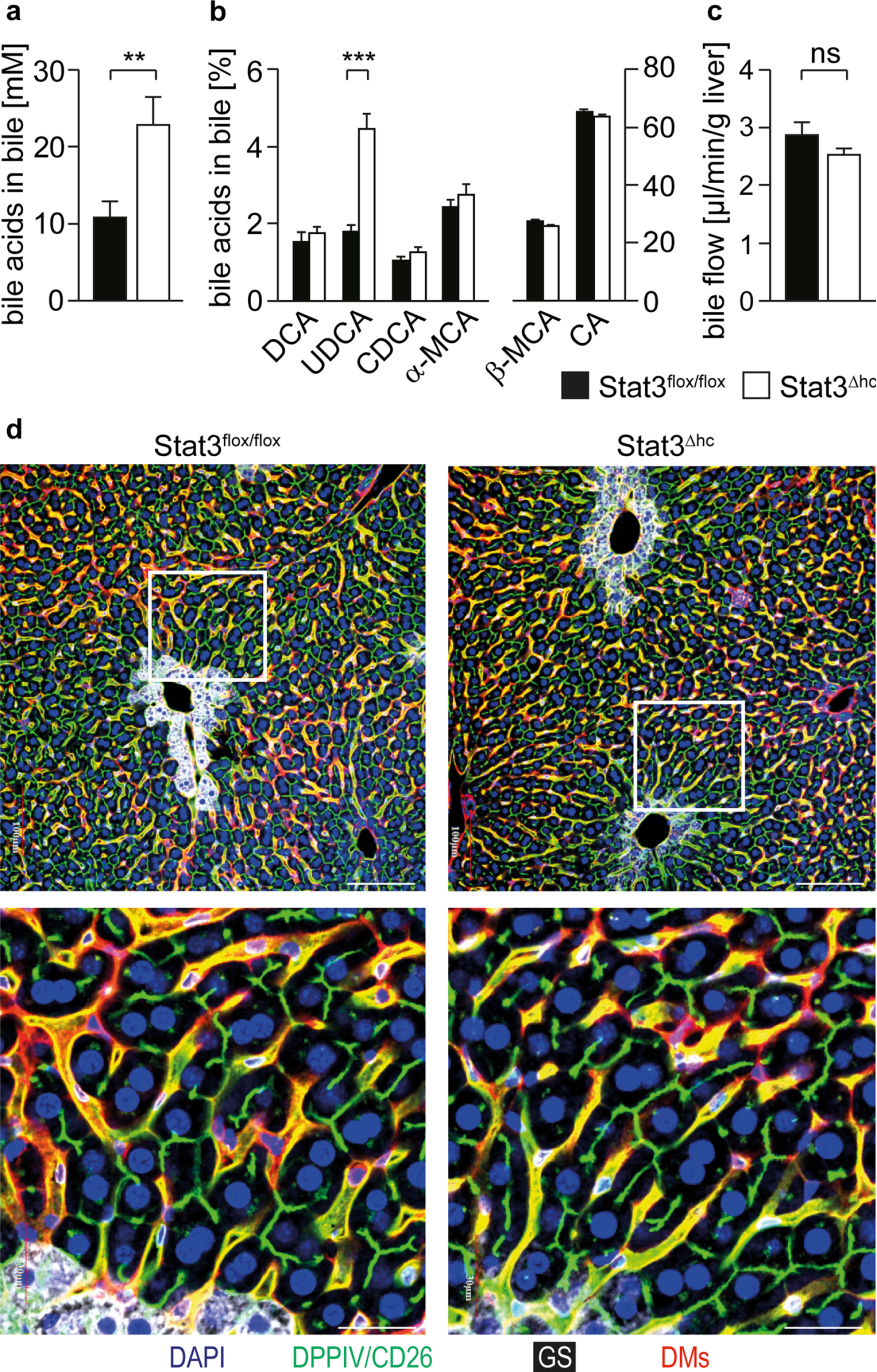
Increased bile acid concentration in the bile of STAT3^∆hc^ mice. **a** The total amount of bile acids was measured in collected bile of STAT3^flox/flox^ and STAT3^∆hc^ mice. *Bars* represent mean data +/− SEM (*n* ≥ 12 animals per genotype; age = 7 weeks). **b** GCMS analysis for bile acid composition in STAT3^flox/flox^ and STAT3^∆hc^ mice. Note that the relative level of UDCA is elevated in STAT3^∆hc^ mice (1.806 ± 0.1683 % *n* = 12 for STAT3^flox/flox^ and 4.468 ± 0.3927 % *n* = 13 for STAT3^∆hc^ mice; *p* < 0.0001). *Bars* represent mean data +/− SEM (*n* ≥ 12 animals per genotype; age = 7 weeks). *DCA*: deoxycholic acid; *UDCA*: ursodeoxycholic acid; *CDCA* chenodeoxycholic acid, *α-MCA* alpha-muricholic acid, *β-MCA* beta-muricholic acid, *CA* cholic acid. **c** The bile flow was measured by gall bladder intubation in STAT3^flox/flox^ and STAT3^∆hc^ mice. *Bars* represent mean data +/− SEM (*n* ≥ 12 animals per genotype; age = 7 weeks). **d** Representative LSM fluorescence micrographs demonstrating normal hepatic microarchitecture in STAT3^∆hc^ mice (*n* = 3 animals per genotype). Formalin-fixed and paraffin-embedded (FFPE) liver tissues were stained for nuclei in *blue* (DAPI), bile canaliculi in *green* (DPPIV/CD26), periportal hepatocytes in *white* (GS), and hepatic sinusoids in *red* (DMs). Scale = 100 μm (low magnification), 30 μm (high magnification)

We have recently shown that STAT3 is a negative regulator of bile acid biosynthesis gene expression [6]. Consistent with increased mRNA expression of Cyp7a1 (cytochrome P450 family 7, subfamily A, polypeptide 1) and Cyp27a1 [6] significantly elevated bile acid concentrations were observed in bile collected of STAT3^∆hc^ (AlfpCre STAT3^flox/flox^) mice (Fig. 1a). GCMS (gas chromatography-mass spectrometry) analysis of bile demonstrated no substantial difference of relative bile acid composition between STAT3^flox/flox^ and STAT3^∆hc^ mice (Fig. 1b), except for increased UDCA (ursodeoxycholic acid) levels in STAT3^∆hc^ mice. UDCA is a primary bile acid in mice, although its synthesis is not clearly defined [17]. No difference was observed in bile flow (Fig. 1c) which was consistent with normal morphology of bile canaliculi (Fig. 1d). These data indicate that STAT3 inhibits production of excessive amounts of bile acids.

### STAT3 protects hepatocytes from bile acid-induced apoptosis

**Fig. 2:**
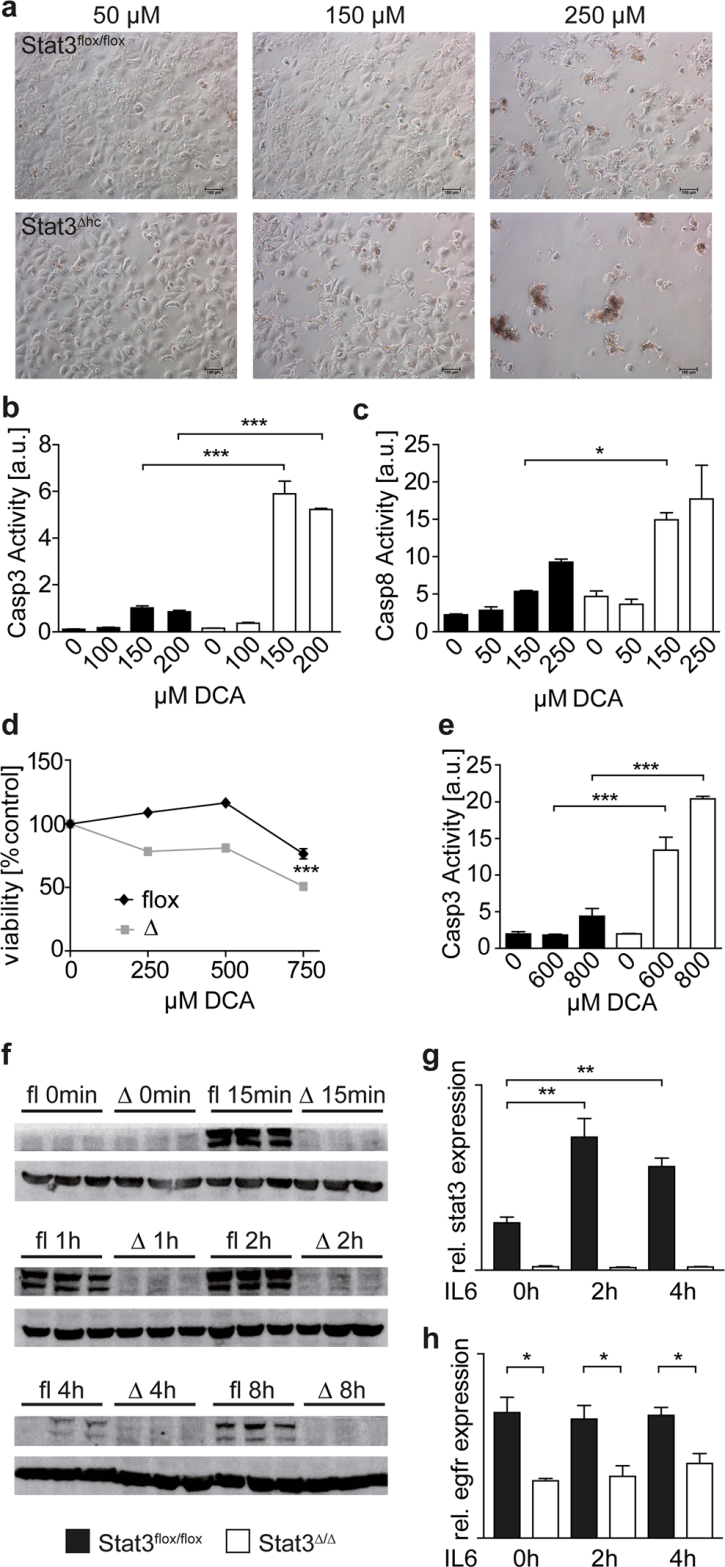
STAT3 protects hepatocytes from bile acid-induced apoptosis. **a** Phase contrast images of primary hepatocytes treated with indicated bile acid (DCA) concentrations for 5 h. Representative images of three biological replicates are shown. **b**, **c** Assays for active caspases 3 (**b**) and 8 (**c**) in cultures of primary hepatocytes after 3 h DCA treatment. Representative analyses of three biological replicates are shown. **d** MTT assay demonstrating reduced viability of immortalized STAT3^∆hc^ p19^ARF−/−^ hepatocytes after 3 h bile acid (DCA) treatment. A representative analysis of four biological replicates is shown. **e** Assay for active caspase 3 in cultures of immortalized hepatocytes after 3 h DCA treatment. A representative analysis of two biological replicates is shown. **f** Western blot analysis for tyrosine-705 phosphorylated pY-STAT3 (*upper images*) in STAT3^flox/flox^ p19^ARF−/−^ and STAT3^∆hc^ p19^ARF−/−^ hepatocytes after IL-6 treatment. The two pY-STAT3 bands represent STAT3α and STAT3ß isoforms. Expression of ß-Actin (*lower images*) was analyzed as loading control. **g** Real-time PCR analysis for STAT3 expression in immortalized hepatocyte cultures after IL-6 treatment. *Bars* represent data +/− SEM of *n* ≥ 3 samples per genotype. **h** Real-time PCR analysis for EGFR expression in immortalized hepatocyte cultures after IL-6 treatment. *Bars* represent data +/− SEM of *n* ≥ 3 samples per genotype

Primary hepatocytes were isolated and treated with bile acids to investigate if elevated bile acid levels in STAT3^∆hc^ mice affect survival of STAT3-deficient hepatocytes in a cell-intrinsic manner. Primary STAT3^∆hc^ hepatocytes were more sensitive to treatment with the bile acid DCA (deoxycholic acid) than STAT3^flox/flox^ hepatocytes (Fig. 2a), and activation of caspase-3 and caspase-8 was observed (Fig. 2b, c). Moreover, we employed loss of the tumor suppressor protein p19^ARF^ for hepatocyte immortalization [19] to establish STAT3^flox/flox^ p19^ARF−/−^ and STAT3^∆hc^ p19^ARF−/−^ hepatocyte cell lines that are reminiscent to primary hepatocytes with respect to morphology and hepatocyte-specific gene expression profiles (data not shown). STAT3^flox/flox^ p19^ARF−/−^ and STAT3^∆hc^ p19^ARF−/−^ immortalized hepatocytes were treated with DCA, and apoptotic cell death was determined. Similar to primary hepatocytes, cell viability of DCA-treated immortalized STAT3-deficient hepatocytes was reduced (Fig. 2d), and caspase 3 was activated (Fig. 2e). These data demonstrate that STAT3 protects hepatocytes from bile acid-induced death in a cell-intrinsic manner. We have recently shown that expression of hepatoprotective EGFR was reduced in STAT3^∆hc^ and STAT3^∆hc^ Mdr2^−/−^ mice. Therefore, we investigated if EGFR expression is reduced in STAT3-deficient hepatocytes and can be induced by IL-6 in a STAT3-dependent manner. Immortalized STAT3^flox/flox^ p19^ARF−/−^ but not STAT3^∆hc^ p19^ARF−/−^ hepatocytes displayed strong tyrosine-705 phosphorylation after IL-6 treatment which was maintained for 2 h (Fig. 2f). Expression of STAT3, which is regulated by IL-6/pY-STAT3 signaling in a positive feedback loop [20], was induced in STAT3^flox/flox^ p19^ARF−/−^ hepatocytes by IL-6 (Fig. 2g). In contrast, EGFR expression was not induced indicating that it is not regulated by canonical IL-6/pY-STAT3 signaling (Fig. 2h). However, EGFR expression was maintained at a constitutively low level in STAT3^∆hc^ p19^ARF−/−^ hepatocytes (Fig. 2h). This suggests that reduced expression of EGFR sensitizes STAT3-deficient hepatocytes to bile acid-induced apoptosis.

### STAT3 protects from cholestatic liver injury via regulation of EGFR

**Fig. 3:**
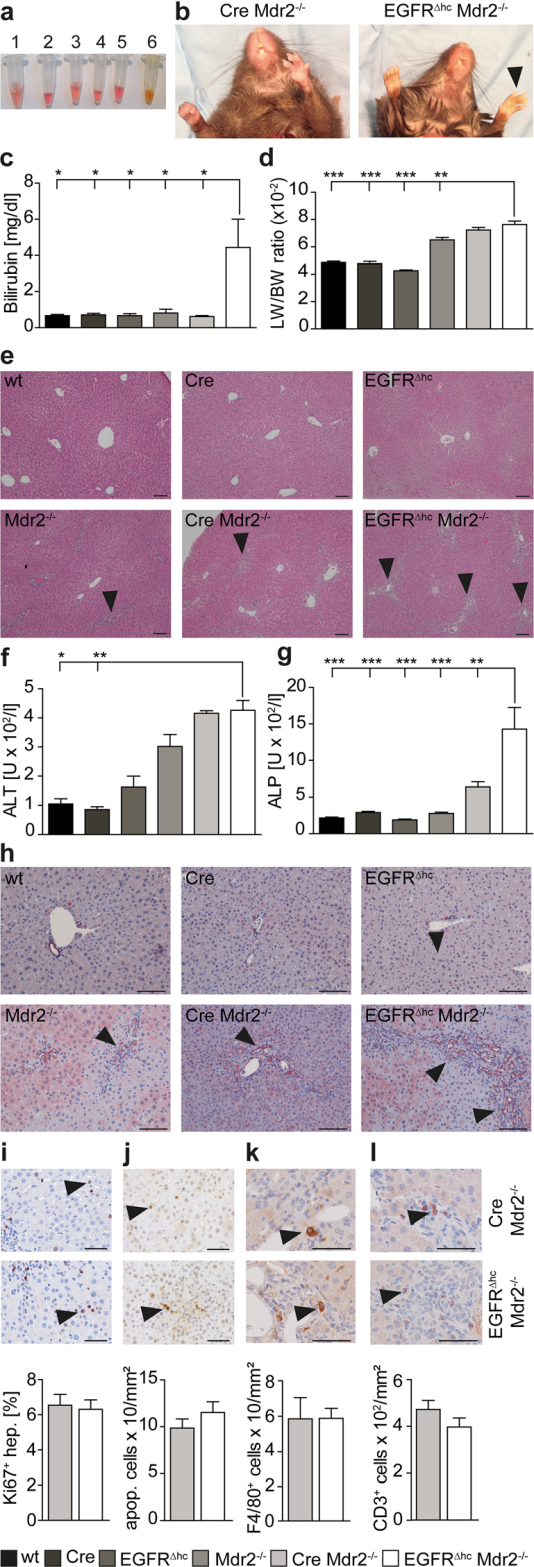
Mdr2^−/−^ mice with liver-specific deletion of EGFR display jaundice and aggravated liver damage. **a** The blood serum of EGFR^∆hc^ Mdr2^−/−^ mice appeared yellow due to high bilirubin concentrations which is indicative for jaundice. *1* EGFR^flox/flox^ (wt), *2* AlfpCre, *3* AlfpCre EGFR^flox/flox^ (EGFR^∆hc^), *4* Mdr2^−/−^, *5* AlfpCre Mdr2^−/−^, *6* AlfpCre EGFR^flox/flox^ Mdr2^−/−^ (EGFR^∆hc^ Mdr2^−/−^). **b** Paws (*arrowheads*) and teeth of EGFR^∆hc^ Mdr2^−/−^ mice appeared yellow due to jaundice. **c** Measurement of bilirubin in the blood serum of indicated genotypes (*n* ≥ 4). **d** Measurement of liver to body weight ratios in indicated genotypes (*n* ≥ 5). **e** H&E-stained liver sections of indicated genotypes showing severe periportal immune cell infiltration in EGFR^∆hc^ Mdr2^−/−^ mice (*arrowheads*). Scale = 100 μm. **f** Measurement of alanine aminotransferase (ALT) in the blood serum of indicated genotypes (*n* ≥ 4). **g** Measurement of alkaline phosphatase (ALP) in the blood serum of indicated genotypes (*n* ≥ 4). **h** IHC staining for Cytokeratin 19 on liver sections of indicated genotypes showing increased cholangiocyte proliferation in periportal areas of EGFR^∆hc^ Mdr2^−/−^ mice *(arrowheads*). Scale = 100 μm. **i** IHC staining for Ki67-positive hepatocytes (*arrowheads*) on liver sections of indicated genotypes and quantitation. *Bars* represent data +/− SEM of *n* ≥ 4 livers per genotype. Scale = 50 μm. **j** Staining for apoptotic cells (*arrowheads*) on liver sections of indicated genotypes and quantitation per liver area. *Bars* represent data +/− SEM of *n* ≥ 4 livers per genotype. Scale = 50 μm. **k** IHC staining for macrophages (F4/80) or **l** T cells (CD3) and quantitation per infiltrated area. *Bars* represent data +/− SEM of n ≥ 4 livers per genotype. Scale = 50 μm

We have shown that activated STAT3 and the closely related STAT5 protein protect from cholestasis-induced liver injury by partly overlapping molecular mechanisms that include regulation of EGFR [6, 21, 22]. Therefore, we employed a genetic approach to evaluate if EGFR is a crucial hepatoprotective factor in cholestatic liver injury and used mice with conditional deletion of EGFR in hepatocytes and cholangiocytes (EGFR^∆hc^). Because recent evidence has suggested that hepatocyte-specific AlfpCre mice display Cre effects could lead to in vivo artifacts [23], we generated all possible genotypes (wild-type, AlfpCre, AlfpCre EGFR^flox/flox^ = EGFR^∆hc^, AlfpCre Mdr2^−/−^ and AlfpCre EGFR^flox/flox^ Mdr2^−/−^ = EGFR^∆hc^ Mdr2^−/−^) of mice and performed biochemical and histopathological analyses of liver injury and fibrosis. Importantly, EGFR^∆hc^ Mdr2^−/−^ mice displayed aggravated liver fibrosis and hepatic damage when compared with control mice. Bilirubin levels were elevated in the serum of EGFR^∆hc^ Mdr2^−/−^ mice (Fig. 3a, b) and they showed signs of jaundice (Fig. 3c). The liver to body weight ratio was increased in EGFR^∆hc^ Mdr2^−/−^ mice when compared with Mdr2^−/−^ mice (Fig. 3d). This was, however, partially due to the AlfpCre transgene because AlfpCre Mdr2^−/−^ mice without EGFR deletion also displayed an increased liver to body weight ratio (Fig. 3d). H&E staining of liver biopsies revealed prominent periportal fibrosis and immune cell infiltration in EGFR^∆hc^ Mdr2^−/−^ mice (Fig. 3e) which was reflected by elevated serum levels of liver damage parameters (Fig. 3f, g) and proliferation of bile ducts (Fig. 3h). However, hepatocyte proliferation (Fig. 3i), apoptosis (Fig. 3j) or numbers of macrophages and T cells in the inflammatory infiltrates (Fig. 3k, l) were not changed in EGFR^∆hc^ Mdr2^−/−^ mice. These data demonstrate that EGFR signaling protects from cholestatic liver injury in Mdr2^−/−^ mice.

### STAT3 protects from cholestatic liver fibrosis via regulation of EGFR

**Fig. 4:**
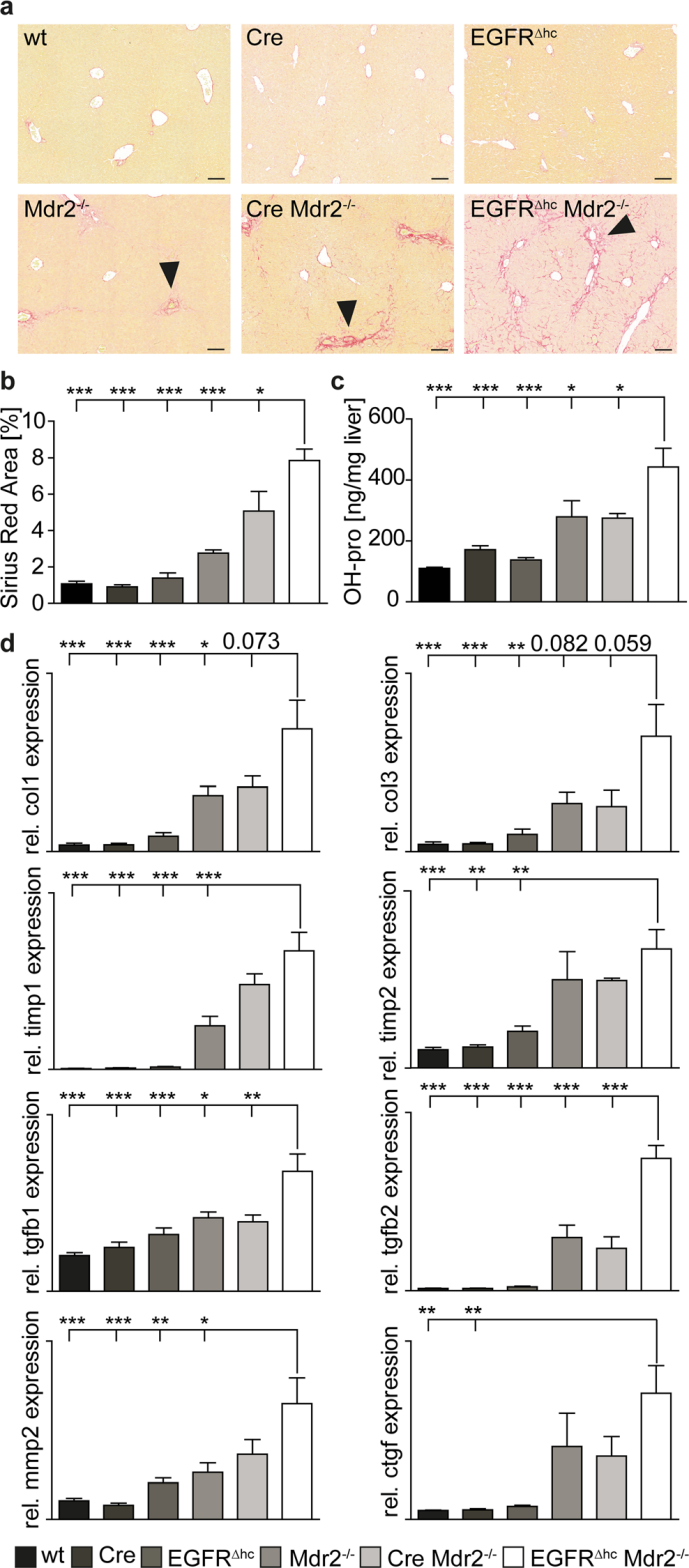
Severe liver fibrosis in EGFR^∆hc^ Mdr2^−/−^ mice. **a** Sirius-red staining on liver sections of indicated genotypes showing increased collagen deposition in periportal areas of EGFR^∆hc^ Mdr2^−/−^ mice (*arrowheads*). Scale = 100 μm. **b** Quantitation of Sirius-red-stained area on liver sections of indicated genotypes using histomorphometry (*n* ≥ 4). **c** Collagen deposition was quantified using biochemical determination of hydroxyproline levels in livers of indicated genotypes (*n* ≥ 5). **d** qPCR for fibrosis markers in livers of indicated genotypes. *Bars* represent data +/− SEM of *n* ≥ 4 livers per genotype. *COL1* type I collagen, *COL3* type III collagen, *TIMP1* tissue inhibitor of matrix metalloproteinase 1, *TIMP2* tissue inhibitor of matrix metalloproteinase 2, *TGFB1* transforming growth factor beta 1; *TGFB2* transforming growth factor beta 2, *MMP2* matrix metalloproteinase 2, *CTGF* connective tissue growth factor

Sirius-red staining for collagen deposition and biochemical measurement of hydroxyproline levels, indicative for collagen deposition, demonstrated aggravated liver fibrosis in EGFR^∆hc^ Mdr2^−/−^ mice (Fig. 4a–c). AlfpCre Mdr2^−/−^ mice were comparable to Mdr2^−/−^ mice demonstrating that aggravated liver fibrosis is due to EGFR deletion but not AlfpCre transgene expression (Fig. 4a–c). qPCR analysis demonstrated increased expression of several key genes implicated in fibrosis in EGFR^∆hc^ Mdr2^−/−^ mice (Fig. 4d). These data demonstrate that EGFR signaling protects from hepatic fibrosis in Mdr2^−/−^ mice.

## Discussion

Hepatic fibrosis is due to chronic liver injury and partially reversible which puts hepatoprotective factors for anti-fibrotic therapies into the limelight. Genetically modified mouse models for liver fibrosis [24, 25] have unraveled effector molecules such as TGF-ß (transforming growth factor beta) [26], PDGF-B [27] (platelet derived growth factor b), PDGF-C (platelet derived growth factor c) [28], or TIMP-1 (tissue inhibitor of metalloproteinase 1) [29], but hepatoprotective factors are not well characterized. We have recently shown that the cytokine IL-6 and the cytokine-inducible transcription factor STAT3 protect from cholestatic liver injury and fibrosis in the Mdr2^−/−^ mouse model for cholestatic liver disease [6]. Both, IL-6^−/−^ Mdr2^−/−^ and STAT3^∆hc^ Mdr2^−/−^ mice showed aggravated liver damage and deposition of collagen in the periportal areas. Gene expression profiling demonstrated that genes for bile acid biosynthesis enzymes were upregulated whereas, EGFR was downregulated in STAT3^∆hc^ and STAT3^∆hc^ Mdr2^−/−^ mice. The mode how STAT3 represses bile acid biosynthesis genes and the implication of known regulators such as FXR-α (farnesoid X receptor alpha) or HNF-4 (hepatocyte nuclear factor 4) [30, 31] has to be determined. We show that blunted repression of bile acid biosynthesis genes at the mRNA levels is reflected by a more than two-fold increase of total bile acid concentrations in the bile of STAT3^∆hc^ mice. Mdr2^−/−^ mice lack a phospholipid pump in the canalicular membrane which prevents formation of mixed micelles between bile acids and phospholipids. The free bile acids are cytotoxic and damage cholangiocytes leading to mild cholestasis and periportal fibrosis [32]. Therefore, elevated bile acid concentrations, as observed in STAT3^∆hc^ mice, are particularly harmful in an Mdr2-deficient genetic background which contributes to aggravated liver damage and fibrosis in STAT3^∆hc^ Mdr2^−/−^ mice.

Aggravated liver damage and formation of bile infarcts have been observed in cholic acid-treated STAT3^∆hc^ mice but hepatocyte-intrinsic effects of STAT3 on cell survival have not been addressed. Here, we show that primary and immortalized hepatocytes, derived from STAT3^∆hc^ mice, were more sensitive to bile acid-induced apoptosis than control hepatocytes. Reduced expression of EGFR, as observed in hepatocytes of STAT3^∆hc^ and STAT3^∆hc^ Mdr2^−/−^ mice, was maintained in immortalized STAT3-deficient hepatocytes. It has been shown that expression of a dominant negative ERBB1 sensitized hepatocytes to bile acid-induced apoptosis. A similar effect was observed after pre-treatment of hepatocytes with the EGFR antagonist Iressa [8–11]. Therefore, our results suggest that STAT3 prevents bile acid-induced apoptosis via positive regulation of EGFR expression in a hepatocyte-intrinsic manner.

The functional consequence of EGFR signaling in cholestatic liver disease was investigated in Mdr2^−/−^ mice lacking EGFR expression in hepatocytes and cholangiocytes. Importantly, EGFR^∆hc^ Mdr2^−/−^ mice phenocopied aggravated liver damage and fibrosis of STAT3^∆hc^ Mdr2^−/−^ mice albeit liver damage and deposition of collagen was less severe. This was reflected by the extended survival of EGFR^∆hc^ Mdr2^−/−^ mice when compared to STAT3^∆hc^ Mdr2^−/−^ mice which died prematurely due to severe jaundice [6]. STAT3 regulates additional hepatoprotective factors such as IGF-1 (insulin like-growth factor 1) [6] which might explain more severe cholestatic liver damage in STAT3^∆hc^ Mdr2^−/−^ mice. Compensatory hepatocyte proliferation and apoptosis were not increased in EGFR^∆hc^ Mdr2^−/−^ mice. Hepatocyte proliferation might be limited by ablation of EGFR which is implicated in liver regeneration after partial hepatectomy [14]. Moreover, bile acid-induced liver damage in Mdr2^−/−^ mice might be rather due to a necrotic mechanism than apoptosis [6]. Our study suggests that activation of EGFR signaling might represent a therapeutic strategy to interfere with cholestatic liver injury and fibrosis. It also emphasizes the need to monitor adverse hepatic effects of EGFR inhibitors in cancer patients that simultaneously suffer from cholestatic liver disease.

## References

[1] Wallace MC, Friedman SL (2014). Hepatic fibrosis and the microenvironment: fertile soil for hepatocellular carcinoma development. Gene Expr.

[2] Wallace MC, Friedman SL, Mann DA (2015). Emerging and disease-specific mechanisms of hepatic stellate cell activation. Semin Liver Dis.

[3] Wang P, Koyama Y, Liu X, Xu J, Ma HY, Liang S, Kim IH, Brenner DA, Kisseleva T (2016). Promising therapy candidates for liver fibrosis. Front Physiol.

[4] Mair M, Blaas L, Osterreicher CH, Casanova E, Eferl R (2011). JAK-STAT signaling in hepatic fibrosis. Front Biosci (Landmark Ed).

[5] Ezure T, Sakamoto T, Tsuji H, Lunz JG, Murase N, Fung JJ, Demetris AJ (2000). The development and compensation of biliary cirrhosis in interleukin-6-deficient mice. Am J Pathol.

[6] Mair M, Zollner G, Schneller D, Musteanu M, Fickert P, Gumhold J, Schuster C, Fuchsbichler A, Bilban M, Tauber S (2010). Signal transducer and activator of transcription 3 protects from liver injury and fibrosis in a mouse model of sclerosing cholangitis. Gastroenterology.

[7] Plum W, Tschaharganeh DF, Kroy DC, Corsten E, Erschfeld S, Dierssen U, Wasmuth H, Trautwein C, Streetz KL (2010). Lack of glycoprotein 130/signal transducer and activator of transcription 3-mediated signaling in hepatocytes enhances chronic liver injury and fibrosis progression in a model of sclerosing cholangitis. Am J Pathol.

[8] Dent P, Han SI, Mitchell C, Studer E, Yacoub A, Grandis J, Grant S, Krystal GW, Hylemon PB (2005). Inhibition of insulin/IGF-1 receptor signaling enhances bile acid toxicity in primary hepatocytes. Biochem Pharmacol.

[9] Qiao L, Studer E, Leach K, McKinstry R, Gupta S, Decker R, Kukreja R, Valerie K, Nagarkatti P, El Deiry W (2001). Deoxycholic acid (DCA) causes ligand-independent activation of epidermal growth factor receptor (EGFR) and FAS receptor in primary hepatocytes: inhibition of EGFR/mitogen-activated protein kinase-signaling module enhances DCA-induced apoptosis. Mol Biol Cell.

[10] Qiao L, Yacoub A, Studer E, Gupta S, Pei XY, Grant S, Hylemon PB, Dent P (2002). Inhibition of the MAPK and PI3K pathways enhances UDCA-induced apoptosis in primary rodent hepatocytes. Hepatology (Baltimore Md.).

[11] Rao YP, Studer EJ, Stravitz RT, Gupta S, Qiao L, Dent P, Hylemon PB (2002). Activation of the Raf-1/MEK/ERK cascade by bile acids occurs via the epidermal growth factor receptor in primary rat hepatocytes. Hepatology (Baltimore Md.).

[12] Alonzi T, Maritano D, Gorgoni B, Rizzuto G, Libert C, Poli V (2001). Essential role of STAT3 in the control of the acute-phase response as revealed by inducible gene inactivation [correction of activation] in the liver. Mol Cell Biol.

[13] Kellendonk C, Opherk C, Anlag K, Schutz G, Tronche F (2000). Hepatocyte-specific expression of Cre recombinase. Genesis.

[14] Natarajan A, Wagner B, Sibilia M (2007). The EGF receptor is required for efficient liver regeneration. Proc Natl Acad Sci U S A.

[15] Smit JJ, Schinkel AH, Oude Elferink RP, Groen AK, Wagenaar E, van Deemter L, Mol CA, Ottenhoff R, van der Lugt NM, van Roon MA (1993). Homozygous disruption of the murine mdr2 P-glycoprotein gene leads to a complete absence of phospholipid from bile and to liver disease. Cell.

[16] Hammad S, Hoehme S, Friebel A, von Recklinghausen I, Othman A, Begher-Tibbe B, Reif R, Godoy P, Johann T, Vartak A (2014). Protocols for staining of bile canalicular and sinusoidal networks of human, mouse and pig livers, three-dimensional reconstruction and quantification of tissue microarchitecture by image processing and analysis. Arch Toxicol.

[17] Sayin SI, Wahlstrom A, Felin J, Jantti S, Marschall HU, Bamberg K, Angelin B, Hyotylainen T, Oresic M, Backhed F (2013). Gut microbiota regulates bile acid metabolism by reducing the levels of tauro-beta-muricholic acid, a naturally occurring FXR antagonist. Cell Metab.

[18] Jamall IS, Finelli VN, Que Hee SS (1981). A simple method to determine nanogram levels of 4-hydroxyproline in biological tissues. Anal Biochem.

[19] Mikula M, Fuchs E, Huber H, Beug H, Schulte-Hermann R, Mikulits W (2004). Immortalized p19ARF null hepatocytes restore liver injury and generate hepatic progenitors after transplantation. Hepatology (Baltimore Md.).

[20] Ichiba M, Nakajima K, Yamanaka Y, Kiuchi N, Hirano T (1998). Autoregulation of the Stat3 gene through cooperation with a cAMP-responsive element-binding protein. J Biol Chem.

[21] Blaas L, Kornfeld JW, Schramek D, Musteanu M, Zollner G, Gumhold J, van Zijl F, Schneller D, Esterbauer H, Egger G et al (2009) Disruption of the growth hormone-signal transducer and activator of transcription 5-Insulinlike growth factor 1 axis severely aggravates liver fibrosis in a mouse model of cholestasis. Hepatology (Baltimore, Md) 51(4):1319–1326 10.1002/hep.23469 PMC2976853 20162728

[22] Stiedl P, McMahon R, Blaas L, Stanek V, Svinka J, Grabner B, Zollner G, Kessler SM, Claudel T, Muller M (2015). Growth hormone resistance exacerbates cholestasis-induced murine liver fibrosis. Hepatology (Baltimore Md.).

[23] Pruniau VP, Louagie E, Brouwers B, Declercq J, Creemers JW (2013). The AlfpCre mouse revisited: evidence for liver steatosis related to growth hormone deficiency. Hepatology (Baltimore Md.).

[24] Crespo Yanguas S, Cogliati B, Willebrords J, Maes M, Colle I, van den Bossche B, de Oliveira CP, Andraus W, Alves VA, Leclercq I (2016). Experimental models of liver fibrosis. Arch Toxicol.

[25] Liedtke C, Luedde T, Sauerbruch T, Scholten D, Streetz K, Tacke F, Tolba R, Trautwein C, Trebicka J, Weiskirchen R (2013). Experimental liver fibrosis research: update on animal models, legal issues and translational aspects. Fibrogenesis Tissue Repair.

[26] Sanderson N, Factor V, Nagy P, Kopp J, Kondaiah P, Wakefield L, Roberts AB, Sporn MB, Thorgeirsson SS (1995). Hepatic expression of mature transforming growth factor beta 1 in transgenic mice results in multiple tissue lesions. Proc Natl Acad Sci U S A.

[27] Czochra P, Klopcic B, Meyer E, Herkel J, Garcia-Lazaro JF, Thieringer F, Schirmacher P, Biesterfeld S, Galle PR, Lohse AW (2006). Liver fibrosis induced by hepatic overexpression of PDGF-B in transgenic mice. J Hepatol.

[28] Campbell JS, Hughes SD, Gilbertson DG, Palmer TE, Holdren MS, Haran AC, Odell MM, Bauer RL, Ren HP, Haugen HS (2005). Platelet-derived growth factor C induces liver fibrosis, steatosis, and hepatocellular carcinoma. Proc Natl Acad Sci U S A.

[29] Yoshiji H, Kuriyama S, Miyamoto Y, Thorgeirsson UP, Gomez DE, Kawata M, Yoshii J, Ikenaka Y, Noguchi R, Tsujinoue H (2000). Tissue inhibitor of metalloproteinases-1 promotes liver fibrosis development in a transgenic mouse model. Hepatology (Baltimore Md.).

[30] Goodwin B, Jones SA, Price RR, Watson MA, McKee DD, Moore LB, Galardi C, Wilson JG, Lewis MC, Roth ME (2000). A regulatory cascade of the nuclear receptors FXR, SHP-1, and LRH-1 represses bile acid biosynthesis. Mol Cell.

[31] Zhang M, Chiang JY (2001). Transcriptional regulation of the human sterol 12alpha-hydroxylase gene (CYP8B1): roles of heaptocyte nuclear factor 4alpha in mediating bile acid repression. J Biol Chem.

[32] Fickert P, Fuchsbichler A, Wagner M, Zollner G, Kaser A, Tilg H, Krause R, Lammert F, Langner C, Zatloukal K (2004). Regurgitation of bile acids from leaky bile ducts causes sclerosing cholangitis in Mdr2 (Abcb4) knockout mice. Gastroenterology.

